# St. Thomas's Hospital

**Published:** 1829-04-01

**Authors:** 


### ST. THOMAS'S HOSPITAL.

[Mr. BURY, reporter.]

### I. ULCERATION OF THE SCROTUM, AND FUNGUS OF THE TESTICLE.

For the particulars of this case we are indebted to Mr. Hunt, clinical clerk to Mr. Tyrrell.

J. Vowels, æt. 31, of an emaciated appearance, was admitted under Mr. Tyrrell, Oct. 2d, having ulceration of the scrotum and fungus of the testis. His habits have been regular, and general health good, independent of syphilis.

Three years ago, he was admitted into this hospital with chancres on the penis, for which he underwent a course of mercury, and, at the end of eight weeks, was discharged, cured; then having a small ulcer on the leg. Some months afterwards he was again admitted, on account of the ulcer, which had extended, and bore a sloughy appearance: he was again salivated, and discharged, some weeks after, incurable. Some months subsequently, he became a physician's out-patient, under whose treatment the mouth was affected by mercury, and the ulcer healed. At this period, 14 months since, his throat became sore. Gargles were used, and decoct. sarsap. with nitric acid, and pil. hydrarg. were taken.

In August last, inflammation and swelling, with considerable pain, attacked the scrotum, and terminated in suppuration. Ulceration occurred at the upper part of the scrotum, through which a small quantity of matter was discharged, and the fungus, the size of a small walnut, protruded.

Has a good appetite—pulse weak—the ulcer, larger than a crown-piece, has a sloughy appearance. The fungus, larger than a walnut, has the appearance of indolent granulations; is hard to the touch, without increased pain on pressure. It seems to arise from the body of the testicle. The pain in it is slight. He has considerable inflammation and ulceration of the soft palate. He was ordered to keep his bed; the part to be supported. To take hyd. oxym. gr. $\frac{1}{16}$, decoct. sarsap. ℥j, in die; opii, gr. j. nocte maneq. garg. hydr. oxy. lot. sodæ chlor. c. capl. lini.

Oct. 6th. The fungus of a more healthy aspect; vin. rubri, ℥vj. quotidie. 9th. Healthy pus secreted from the scrotum—more pain in the throat—the ulceration slightly extended. He has cough, with dark viscid expectoration. Pergat; sine hydrarg. oxymur. 13th. Throat better—fungus less, but the ulcer spreads. A mutton chop daily. 18th. Improvement on 13th; The edges of the ulcer are, in many places, spotted with dark-coloured sloughs. Porter, ℥j. daily. 27th. Improvement has continued. The fungus is nearly level with the body of the testicle. Edges of the ulcer granulating kindly; on some parts cicatrization has formed—throat also much improved. Wine, porter, and mutton chop daily.

Nov. 10th. The fungus scarcely perceptible—the ulcer considerably decreased. 18th. No remains of the fungus—there is still very trifling ulceration of the integument of the scrotum, of a healthy character.

This is the third instance in which Mr. Tyrrell has succeeded in curing these fungous growths from the testicle by constitutional remedies, and the same mild local treatment.

### II. LITHOTOMY.

*Case* 1. Thomas Gash, æt. 5, admitted, 2d Oct. under Mr. Green. The person who accompanied him to the hospital, stated that he had been troubled with the symptoms of stone from the time that he was weaned; that he had had a cough for twelve months, till within a fortnight of his admission; and that he had always been considered delicate, but had improved latterly, and observably gained flesh. His sufferings have been severe, and the prepuce is a good deal elongated; sleep is generally good, and appetite also.

Oct. 10th. To day Mr. Green performed the operation, and removed a large-sized calculus from the bladder. 11th. Urine has flowed freely during the night, and up to 8 o'clock this morning, since which the secretion has been scanty—slight abdominal tenderness—pulse quick and full—skin hot—face flushed. Hirud. xij. abdom. Mr. G. observed that, by the early adoption of depletion, there was every probability that the inflammatory action would be cut short. 12th. Pulse quick, but not full—slight tenderness of abdomen continues—skin temperate—no flushing—no thirst—urine passes in small quantity—he takes but little drink. Rep. hirudines; ol. ricini, ℥ij. 13th. Urine is not secreted so plentifully as is usual —no inclination to drink—abdomen appears tender on pressure—pulse quick—sleeps well—bowels opened four times. 15th. The flow of urine has become more abundant—the wound looks remarkably well, and there is no tenderness whatever.

18th. Discharge of urine is plentiful, and some has passed per urethram—meat allowed—bowels require to be opened by castor oil.

*Case* 2. Edward Harrison, æt. 11, admitted under Mr. Green, Oct. 18th. Has been the subject of stone in the bladder from his infancy, but is not at all emaciated, having, on the contrary, a ruddy countenance, and every appearance of health. Was sounded by Mr. G. who detected one calculus readily, and was inclined to think that a second existed.

24th. His bowels having been twice freely acted upon by house-medicine, and the ordinary diet enforced, lithotomy was performed, and a round and large calculus extracted. 25th. The patient has, on the whole, passed a good night, but awoke, at intervals, crying, on account of the coldness of the linen, wetted by his urine, which has flowed freely, a little having escaped per urethram—he has some tenderness of abdomen, in hypogastrio especially—pulse quick and full —face flushed—skin hot, but not dry—tongue white, and a little furred. V.S. brachio. 27th. On account of increase of pain and tenderness in the afternoon, twelve leeches were applied yesterday. Pulse to-day quick, but compressible—tenderness continues, with a little pain, on pressure, in left hypochondrium—face is flushed—urine has passed in plenty—bowels are opened by medicine. Rep. hirud. enema commun. stat. 29th. All inflammatory symptoms removed.

Nov. 12th. The improving state of the patient has been uninterrupted. A discharge of purulent matter, both more in quantity and of different quality from the ordinary secretion of a granulating wound, has come from the perinæum during the last week. This, Mr. Green supposes, may probably be the effect of suppurative inflammation having occurred in some part of the cellular tissue, as was indicated by the pain more particularly felt in the left hypochondrium for a few days subsequent to the operation. If such be the case, he thinks the favourable termination of the inflammation is owing to the *early* adoption of antiphlogistic measures; the advantages of which were very conspicuous in this, as well as the preceding case.

### XXXIII.

### ST. THOMAS'S HOSPITAL.

[Mr. Bury, Reporter.]

### I. PURULENT OPHTHALMIA SUCCEEDING TO GONORRHŒA.

*Case* 1. W. Webb, æt. 34, admitted under Mr. Travers, Oct. 30th, 1828, with purulent ophthalmia of both eyes, which had commenced three weeks previously, and about seven days after a gonorrhœal discharge had appeared from the urethra. The lids of both eyes were approximated, considerably swollen, and an abundant purulent secretion came from between them.

About three weeks ago, whilst he was employed in breaking stones, and shortly after micturition, a particle of stone struck his left eye, which led him immediately to rub it with his fingers. Pain, and swelling of the eye-lids took place, he says, instantly, and the inflammation ran to such a degree as to destroy all vision of that eye in the course of an hour. The right eye became affected in the same manner a week afterwards. From the commencement of the attack he has experienced very severe pain in the ball, lids, temples, and occipital region, and also that sensation of minute extraneous particles on the conjunctiva so frequently observed in inflammation of that membrane, arising from distention of the conjunctival vessels; and these symptoms now continue, though much less intense than they have been, in the right, but not at all in the left eye. He has made use of no application to the eyes, and has taken only opening powders.

Upon separating the lids of the left eye, which was done with some difficulty, the lens immediately escaped, with a portion of the vitreous humour, through the cornea, which was in a sloughing condition, although no force whatever was used at the time. There was slight chemosis of the conjunctiva of the right eye, with imperfect vision and great intolerance of light; but the cornea appeared clear. Pulse frequent, but not full. Tongue white, and rather dry. Appetite not much impaired. The quantity of urethral discharge was not profuse, it having, he said, diminished upon the occurrence of inflammation in the eye to nearly one half. Ardor urinæ still remains. Ordered, small doses of magn. sulph. in infus. ros. co. Pil. sapon. c. opio, gr. iv. o. n. Milk diet. Hirudines vj. tempori dextro, et lotio plumbi. Empl. lyttæ pone aur. dextram. A bread poultice to the left eye.

Nov. 3d. Pain of head somewhat less, and also of both eyes, which appear much in the same state. Pulse a little quick, and small; tongue whitish. ℞. Sodæ carb. gr. v. Pulv. cinchonæ, ℈ss. ℳ. ft. pulv. 4tis horis sum. Rep. hirudines. An alum lotion substituted for that of lead.

7th. Discharge from the conjunctivæ is less, and the patient's sufferings likewise—no pain now in head or temples—redness of right conjunctiva not so vivid—sight more indistinct. Leeches have been twice applied since the 3d to the temples and eyelids; the pain of right eye is alleviated for about two days by each application—pulse soft—desire expressed for animal food—bowels have a tendency to constipation, but are opened by house-medicine. Pulv. cinchonæ, ʒss. Sodæ carb. gr. v. 6tis horis. Beef infusion twice daily. Rep. hirud. 3tiis auror.

12th. Pain of eyes and head very trifling, being greatly relieved by the leeches—discharge from the eyes becoming less—that from urethra has gradually decreased since his admission—chemosis of the right eye increases, and the cornea appears dull.

17th. Conjunctiva of right eye more swollen and prominent—in other respects the state of eyes remains the same. On the 15th a blister was ordered to the nucha, to be kept open by savine cerate; the lead lotion was also resumed. Bark and soda omitted. ℞. Quinæ sulph. gr. j. Magnes. sulph. ʒj. Inf. ros. co. ℥x. t. d.

22d. For the last three days he has been able to open the right eye, the vision of which, he says, is rather more distinct, but ulceration has taken place over the greater surface of the cornea. The purulent discharge has greatly diminished from both orbits; from the right there is a free watery secretion, apparently lachrymal—no pain—great improvement in other symptoms.

Dec. 1. Puriform secretion has ceased, but sight is very little improved.

13th. Ulceration of cornea in great measure removed by a lotion of argenti nitras, in consequence of which the sight is rendered more distinct.

20th. The cornea more clear, and has a cluster of red and varicose vessels ramifying upon it. The iris is irregular, and adherent in some parts to the former membrane.

28th. There is less irregularity of the pupil, and the circle of vessels is diminishing—patient says his sight seems gradually improving.

1829, Jan. 10th. Vision a little increased—some deep opacity of the cornea opposite the pupil appears to prevent better sight—drops of the nitrate still used.

Jan. 24th. No alteration, according to the patient's account, in the powers of the organ—the transparent cornea has lost a little of its convexity. The ball is considerably sunken within the orbit, as if from the cushion of fat at its back part having become absorbed. Some firm adhesions have become established between the conjunctiva of the lower lid and that opposed to it covering the sclerotica. The iris is generally adherent anteriorly, and is of course devoid of mobility. In a few days the man left the house.

*Case* 2. Robert Suttle, æt. 29, received into the hospital on the 27th Nov. 1828. Was attacked about a month ago with gonorrhœa, which now continues, but without any inflammatory symptoms, and the discharge being very inconsiderable. Inflammation commenced in his right eye eighteen days ago, accompanied with the symptoms of purulent ophthalmia in a mild degree. He said that he was himself ignorant of a direct cause of these symptoms, although Mr. Travers, who saw him before he applied at the hospital, distinctly traced their origin to the local application of matter from his urethra. After an interval of three days the left eye was similarly attacked. He lost no time in applying for advice, and underwent antiphlogistic treatment, general and local, with very great advantage. This improvement was, however, completely negatived by his travelling about ten days since from Southampton to London on the outside of a coach, when the weather was rainy and the wind cold. The inflammation of both eyes was materially aggravated; the lids of the right swelled to a great extent, and the small power of vision which the organ had before possessed was now entirely destroyed; the sight of the left eye, which, he said, was pretty good before, was in like manner lost. Very severe antiphlogistic means appear to have been judiciously put in practice at this time by a practitioner in town. He was bled repeatedly, cupped from the temples, and leeched; and had blisters, &c. with free purgation. The severity of the renewed attack was thus lessened, and the following were the principal points of the case on the day of admission: the eyelids were not much swollen, and the pain was not acute—he could see but very imperfectly, and could not open either eye—the conjunctivæ of both eyes and palpebræ were swollen, of a deep red colour, and of irregular surfaces—purulent discharge had been abundant, but was not now so great. The secretion from the urethra had observably diminished since the eyes had been attacked, and the patient thought more rapidly during the last ten days. Patient slept but little; pulse quick and small; bowels not open; tongue white. Considering the disease to have passed into the atonic stage, Mr. Green prescribed as follows:—Quinæ sulph. gr. ij. ter die. Sulph. alum, gr. j. Aquæ, ℥j. to be injected over the eyes. Hirud. ij. sing. palpebris. Pul. rhæi c. hydr. stat.

Nov. 29th. Leeches produced considerable relief of pain and swelling; he could partly open his eyes yesterday. Bowels opened once only. Pulv. scamm. c. hydr. gr. xv. Hirudines iv. sing. palpeb. Sulph. alumin. gr. iss. ad aquæ, ℥j. Empl. lyttæ nuchæ.

Dec. 2d. Both corneæ in a sloughing condition—the pain is less.

16th. Patient's strength and appetite are beginning to return; he asks for some porter; pulse has a little more strength, and is not quite so frequent. Little alteration is perceptible in the organs of vision. Quinæ sulphat. gr. iij. ter quotidie.

23d. Sloughing has advanced—increased dulness in portions of corneæ not destroyed, and chemosis undiminished. He has been seen by Mr. Travers. Very slight hopes of the recovery of vision are entertained.

1829, Jan. 7th. Inflammation has greatly subsided, but the appearance of both corneæ is very unfavourable; these being flattened, having lost all transparency, and being covered with irregular lines of deposit between their laminæ. There are some marks of pupils, of most unnatural figure. Light can be just discerned.

20th. Some improvement of sight has taken place, allowing him to distinguish the motions of his hands. The natural prominence of both eyes now no longer continues.

30th. There is very little prospect of any change in the state of the corneæ or in that of vision, the great disorganization which is produced being beyond further relief. Considerable vascularity of conjunctiva is to be observed in each eye. The quinine has been persevered with internally, and alum lotion externally.

As it is admitted that the form of purulent ophthalmia, connected with the disease of gonorrhœa, is the most dangerous and rapid in its progress of any, so, in its early stages, the most vigorous treatment is called for, and by nothing but this have we any chance of arresting its destructive effects. But after this stage has terminated, and with it the violent inflammatory symptoms, the inflammation not infrequently assumes a *passive* character, as in both of the instances we have related, and, under such circumstances, a different mode of treatment is necessarily required. And the evidence of these two cases would appear to shew that an effectual plan of relief during the atonic stage consists in giving *general* support to the system by bark or quinine, and in removing the *local* congestion by leeches or cupping; the former of these will be usually found to answer best, as they can be applied directly to the loaded vessels on the surface of the conjunctiva. The preservation (though by no means complete) of the cornea in the first instance before us, was certainly fortunate, for "after the first, or acute stage, is over, we are not in general able to save this membrane from destruction by sloughing."

In each of the above cases the disease seems to have originated from the application of gonorrhœal matter to the eyes. Mr. Travers and Mr. Green entertain different opinions touching the cause of this destructive ophthalmia. The former gentleman states from experience that no case has ever fallen under his notice, in which he could not trace its origin to the contact of matter, and, therefore, this he believes to be the cause of the disease. And so minute a quantity of the morbid matter is sufficient to give birth to the affection, that he has known some of his dressers, who, we may fairly presume, did not act altogether unguardedly during their operations, to be attacked with it, and ultimately deprived of sight, from the reception of a small portion of the matter upon their hands or fingers, of which they were quite unaware, when employed in cleaning the eyes of ophthalmic patients with a syringe. On every occasion the unfortunate gentlemen acknowledged that they had applied their fingers to their eyes in rubbing them, or some other mode, either at the time of using the syringe, or shortly afterwards. In a clinical lecture upon the second case, Mr. Green mentioned that a *sympathetic* action might take place between the mucous membrane of the eye and that of the urethra, when this was affected with gonorrhœa, and a secretion be poured forth from the former similar to that from the latter. Discharges of an unnatural kind are very often produced in parts from sympathy with others at a distance, and this relation of sympathy is especially maintained in membranous surfaces of the same class. In gouty affections, Mr. G. observed, there sometimes occurs a very profuse discharge from the urethra not dissimilar to the gonorrhœal; and taking the number of uncleanly persons who have gonorrhœa into consideration, we might expect that the frequency of this form of ophthalmia would be much greater than it is, if the disease were produced by application of the poisonous matter. We leave it to others more experienced than ourselves to form their own judgment upon this point.

### II. Extraordinary and dangerous Symptoms from drinking Porter.

On Thursday, January 15th, two men, between 20 and 25 years of age, were taken into the hospital about 12 o'clock in the day, both under the same circumstances, and affected with similar symptoms.

They were in a state of complete insensibility, and had lost all power of voluntary motion and all sensation. Their pulse were at first weak and very slow.

The men were quickly carried into two different wards. In the course of a quarter of an hour the pulse had become, in both patients, full, hard, and labouring, between 40 and 50 ; respiration difficult, and somewhat stertorous ; pupils rather contracted, and motionless ; jaws firmly closed ; profuse and cold perspiration on the body, which was greatest and of longest duration upon the face ; no sickness or vomiting ; no paralysis of the sphincters ; face and trunk were said by some friends of the patients to be swollen. Venesection from the arm was immediately practised to a large amount in both instances.

In the one case cupping was also performed at the back of the neck, and emetics of sulphate of zinc given repeatedly, but ineffectually. Croton oil was then administered in two or three doses, but we believe no evacuation was produced from the bowels thereby. The volume of the pulse was reduced by these measures, it became more frequent and natural, the extremities and surface in general were diminished in temperature, and a state of exhaustion had ensued by 3 o'clock, p. m. An enema of castor oil was given, and mustard sinapisms applied to scrobiculus cordis ; copious dilution of the contents of the stomach by warm water got down at short intervals. No return of sensibility was, however, produced, and stimulants appeared to be indicated ; brandy and water were accordingly exhibited. The stomach was emptied of its contents by the tube and syringe at 4 o'clock, and the stimuli of brandy and ammonia afterwards thrown into it. At 7, p. m. he had revived a little, was able to answer questions, and his dangerous symptoms had begun to disappear. The temperature of the body was raised, and the pulse had risen from the stimuli. This amendment continued during the evening, with the gradual return of volition and consciousness, and on the next day the man said he was quite well, independently of the effects of the treatment adopted.

In the other individual the greater portion of the contents of the stomach was drawn off as soon as the instrument could be made to act. The tube, of the usual length, was not sufficiently long to effect complete evacuation of the stomach. Cold was applied to the head after the stomach-pump was withdrawn. It was also used in the preceding instance. A degree of coldness of the extremities and general surface of the body, with an alteration in the pulse, indicated considerable reduction of the vital powers by 2 p. m. The breathing was a good deal relieved, and the pupils had some slight motion. Stimulants were now administered, and with the best effect. About 4 o'clock the patient rallied, and gradually recovered from that hour even more rapidly than the former.

It appeared that both of these men, having been unemployed for some time, had, on the above day, recommenced labour *by task-work*, at the new London Bridge ; that they had worked hard from 7 o'clock in the morning to half-past eleven, when they relaxed for a few minutes, considerably heated and fatigued, and drank, most probably at one draught, some warmed porter with ginger in it, (one account stated that this was floating on the top of the fluid in their cups,) the one a pint, and the other half a pint. The porter was poured out of a large vessel which was full, and they were the first who drank of it. Some of the persons (fellow labourers) who accompanied them to the hospital, declared that others had drunk after them of the porter in the same vessel without suffering any inconvenience ; but one or two others, on whom more reliance was to be placed, denied the fact of any one else having tasted the same porter. In the space of ten minutes after having drunk the porter, we learnt from the one man after his recovery, that he observed the other to be giddy and complaining of general malaise, but almost immediately after this he was seized himself with giddiness, and would have fallen down, had not another man come to his assistance. About 20 minutes from the time they drank, they were both perfectly insensible, and we may suppose, in nearly the same condition as that in which they were admitted. Both men informed us, when recovered, some days afterwards, that they had perfect recollection of what transpired for a quarter of an hour after having drunk their draught, but from that period to their returning to sensibility at the times specified they remembered nothing.

A most interesting point for consideration, and one important to ascertain if it were possible, depends upon what gave rise to the anomalous, and truly dangerous (for no one who saw the cases entertained other than unfavourable opinions of their termination) symptoms here presented. That they did not arise from the effect of the porter *perse*, we do believe, as it was obtained from a house of respectability, and many other persons had previously, and have, no doubt, since drunk of the same. The general impression upon the minds of those acquainted with the particulars of the cases appears to be this, that some poisonous and intoxicating article was intentionally (by way of joke) thrown into the cups out of which the men drank, by some of their companions, mixed with the ginger alluded to. Some facts give a very strong colouring to the probability of this, such as the affirmation by the other workmen, that others had drunk out of the same large vessel with impunity, which was again contradicted; the circumstance of the substance floating on the surface of the liquor, as if not thoroughly mixed with the whole; the production of equally severe symptoms with the other, where *half* the quantity of porter was taken in the one instance, supposing that the same quantum of noxious ingredient was made up for both; and, as the unfortunate men were Irish, newly engaged, and most likely strangers to the rest, these might contemplate a joke of the kind, any thing but a bad one. But nothing poisonous, or unusual, has been detected in the fluids extracted from the stomach of the subject of the second case, by Dr. Burton, our Lecturer on Chemistry, though doubtless great pains and care have been observed in the analysis; and in the absence of such proof we cannot give our undivided assent to the above supposition regarding the cause of the symptoms induced. Might not the simple fact of the men having taken an *instantaneous* stimulus, when exhausted, account for all the phenomena produced by this porter, especially as it was conjoined with ginger? We know from experience that the energy of the brain and nervous system, reduced to a low ebb, may be *occasionally overcome* by the *immediate* exhibition of stimuli, these being too great for its then impaired condition, and from *such* a state these two men appear, in our minds, not to have greatly differed when they drank the porter.

*Note by the Editor.*—These cases are very mysterious, as to the cause of the symptoms. Had the porter been cold, the weather hot, the fatigue great, and the draught copious, the phenomena might fairly be attributed to the sudden impression of cold on the stomach, by which many lives are annually lost, especially in the southern states of America. But a draught of half-a-pint of *warm* porter, with *ginger*, however great the state of fatigue on the part of the drinker, affords no probable solution of the mystery. The Editor is, therefore, inclined to think that some narcotic substance had been put into the porter by some means or other; and as this would, of course, be of a vegetable kind, the detection afterwards would be very difficult by means of chemical analysis. Was it the berry of the deadly night-shade? Or was it (which is more likely) powdered tobacco-leaves? It is highly probable that a sufficient dose of the *latter* would produce the phenomena in question, though the intention might have only been to make the men sick.

---

### XXXIV.

ST. THOMAS'S HOSPITAL.

[MR. BURY, REPORTER.]

TALIACOTIAN OPERATION FOR A NEW NOSE SUCCESSFUL.

Thomas Wilkinson, twenty years of age, was taken into the theatre, Oct. 17th, 1828, with the following appearances and history.

The nose had been completely destroyed by ulceration, there being left neither alæ nasi, septum, vomer, nor nasal bones. A triangular opening was thus formed into the interior of the nose. The edges were healed, firm, and healthy. In looking into the throat, we perceived portions of the uvula and tonsils in like manner lost, but the palate was sound. The sense of smell was slightly impaired, and the voice, as was to be expected, had a nasal sound.

The patient traced the origin of this affection to a gonorrhœa, which appeared upon him about two years ago in Lisbon, for which he was profusely salivated for three months. From this period, ulceration commenced in the throat, and, not long afterwards, attacked the nose, where it continued to extend, until it arrived at the point of destruction now visible, without the application of any remedial measures, or, at least, such as could arrest its progress. He had attributed the ulceration of the nose to a blow which he received from a fall, at the time he was under mercurial action; but this, together with the questions of a syphilitic, mercurial, or *their* compound origin, has little to do with the present purpose.

Mr. Green considered that, as the man's health had been restored by alterative medicine and nutritious diet, the present afforded a favourable opportunity for the Taliacotian operation. He, therefore, commenced by making two grooved lines, parallel to the sides of the triangular opening, whereto he might affix the alæ nasi and sides, and another, from one-half to a third of an inch long, at the centre of the base connecting these two sides, whereto the septum (columna) might be applied; so that the new nose should be larger than, and external to, the original, circumscribing it at the distance of about a quarter of an inch. Mr. G. had availed himself of an appropriate model of a nose, by which he might judge of the quantity of integument necessary, and from which he had marked out the same on the forehead. There was, consequently, drawn a circular line, interrupted above by a process, of half an inch in length and three lines in breadth, answering to the natural columna nasi, and below by another, about the same length, and five lines broad, constituting the isthmus, or that narrow slip whereby the vitality of the separated skin was to be maintained, until the necessary healing processes should be established in its new situation. The situation of this portion, described by the circle, was vertical, that is to say, the centre corresponded with the median line of the face. The margin of this circle was followed by the knife, and the whole integument included detached from above downwards, as far as the isthmus, and brought down by a twist to be adapted to the furrowed lines previously mentioned. Being placed with its raw margins upon these grooves, the skin was then moulded to the form of a nose, its dorsum supported by small pieces of lint. Four sutures, on either side, were carefully introduced by curved needles, passed from without inwards, behind the outer edges of the grooves, and these being turned back, from within outwards, embraced the posterior and raw edges of the sides of the new nose. The stitches being tied, the part was securely fixed. A small roll of charpie was inserted between the columna and ala on each side, to preserve the cavities of the nostrils; and some small long pads of the same were placed a little externally to the furrowed lines, being retained there by narrow transverse slips of adhesive plaister, in order to steady the part, to prevent the integument from retiring, and to give firmness to the raw surfaces in contact. A piece of flannel was laid over the nose, for the preservation of its temperature. The wound of the forehead was dressed with charpie and simple ointment. Two or three vessels bled freely during the operation, one, particularly, at the internal angle of the right eye, which was not taken up, but produced considerable inconvenience afterwards. The operation lasted an hour and a half, and was borne with extreme courage. The patient felt exhausted at the termination, and, in truth, he could not have felt otherwise.

On the third day, nothing particular had occurred since the operation. He had slept well—his bowels had been attended to—and he was kept upon slops. The dressings were with care removed—the face and new nose were a good deal swollen—there was a thin, but not unhealthy, discharge from the edges—the sutures did not appear to excite irritation, and the parts were still in good apposition—some portion of the charpie was withdrawn from the nose. That this possessed sufficient vitality was evident, from the circumstance of blood being ejected, per saltum, from a vessel on one side of it, corresponding to the ala. On the right side, some blood (from the hæmorrhage on the evening of the operation) had become interposed between the raw edges, and had prevented perfect agglutination there. No lint, or other kind of support, was placed within the nostrils at this dressing.

Oct. 22d (fifth day.) The tumefaction and thickening of the nose were undiminished, and, from this cause, the newly-formed part had, to appearance, extended in every direction beyond the furrows to which it was adapted; this had taken place at the lower part, so that the septum was bordering on the margin of the upper lip. A degree of adhesive inflammation, as Mr. Green remarked, seemed to have occurred in the whole integument implicated, producing this swollen and thickened condition. All the sutures were cut away, the upper one of the left side excepted, but very little ulceration having taken place from their presence. On the left side of the nose, adhesion appeared perfect. At the upper and lower portions of the right side there was no union, nor at the columna; but, except at the latter part, the raw surfaces were opposed to each other and granulating; and it was thought that the granulations might be made to adhere, by being firmly kept in contact. This was attempted by means of lint and strapping. There was a tolerably free discharge from the cavity of the nostrils; some more pieces of charpie were hence extracted. Patient complained only of debility. Had taken beef-infusion, soup, &c.

25th. The state of patient has been favourable since the 22d. The parts have been dressed daily. Tumefaction has considerably subsided, and the nose is of quite natural colour. On the right side, the granulations appear healthy, but secrete a rather offensive matter. The remaining suture on this side was removed, and the dressings applied as before.

Nov. 1. Patient complains of nothing, having gained strength from the allowance of quinine and two eggs daily. A small slough has separated from the right ala—there is purulent matter discharged from the cavity somewhat abundantly. The union on the left side of the nose is firm and complete; that on the right is extended downwards, and there remains now but a very small surface at the ala to adhere. There is no union at the septum. The size of the part has greatly decreased, but is not yet natural. The bowels require to be acted upon by medicine. Mutton chop daily, to be eaten of a pulpy consistence. Ol. ricini, ℥iij. p. r. n.

12th. The part is still further diminished, and is firmly fixed. The right ala is united, except at one small point, where there is a luxuriant granulation, which was touched with cupri sulphas. The septum has a slight turn to the left side. Wound of forehead has, at every period, been very healthy, and, if much contracted, the cupr. sulph. is applied to its edges. Patient's bowels inactive.

Dec. 16. Ever since last date the state of the parts has progressively improved. Cicatrization is complete in the wound made on the forehead as well as along the adherent edges of the new nose. A slight blush of inflammation, followed by ulceration, has lately appeared in the latter situation on one side. The nose itself is considerably flattened and depressed at its lower part. There are no longer visible any marks of the skin having been twisted at the isthmus in the operation.

1829, Jan. 20. From the liberal diet the patient has been allowed, he has of late suffered some derangement of the digestive function and of the vascular system, arising apparently from no other cause than repletion. He was feverish, had severe catarrhal symptoms, and some inflammation around the tonsils and at the back of the pharynx, but no ulceration was observed. Merely by lowering his diet and by quietude, these symptoms have now disappeared. The sense of touch in the nose is imperfect; its appearance is unchanged. A very vascular cicatrix remains on the forehead.

Feb. 21. No further change has ensued. It is, we believe, the intention of Mr. Green to form another transverse groove for the attachment of the septum, similar to that made in the original operation. We conceive that no difficulty will be experienced in procuring the adhesion of such small surfaces as it will be necessary to make raw for that purpose. There will be no necessity from the circumstance mentioned above for the division of the skin at the isthmus, as was at first supposed.

